# European Respiratory Society Research Seminar on Preventing Pediatric Asthma

**DOI:** 10.1002/ppul.27401

**Published:** 2024-12-03

**Authors:** Jonathan Grigg, Benjamin Barratt, Klaus Bønnelykke, Adnan Custovic, Markus Ege, Christian Pasquali, Oscar Palomares, Seif Shaheen, Milena Sokolowska, Donata Vercelli, Rick Maizels, Erika von Mutius

**Affiliations:** ^1^ Institute of Asthma and Allergy Prevention, Helmholtz Zentrum München, German Research Center for Environmental Health Neuherberg Germany; ^2^ Blizard Institute Queen Mary University of London London UK; ^3^ MRC Centre for Environment and Health, Environmental Research Group, Imperial College London London UK; ^4^ Copenhagen Prospective Studies on Asthma in Childhood Copenhagen University Hospital Copenhagen Denmark; ^5^ National Heart and Lung Institute, Imperial College London London UK; ^6^ Dr von Hauner Children's Hospital Ludwig Maximilian University; Institute of Asthma and Allergy prevention, Helmholtz Centre Munich; Comprehensive Pneumology Center Munich (CPC‐M), German Center for Lung Research Munich Germany; ^7^ Department of Preclinical Research OM Pharma SA Meyrin Switzerland; ^8^ Department of Biochemistry and Molecular Biology School of Chemistry, Complutense University of Madrid Madrid Spain; ^9^ Wolfson Institute of Population Health Queen Mary University of London London UK; ^10^ Allergy and Lung Health Unit, Melbourne School of Population and Global Health The University of Melbourne Victoria Australia; ^11^ Swiss Institute of Allergy and Asthma Research University of Zurich Davos Switzerland; ^12^ Department of Cellular and Molecular Medicine The University of Arizona Tucson Arizona USA; ^13^ Asthma and Airway Disease Research Center The University of Arizona Tucson Arizona USA; ^14^ The BIO5 Institute The University of Arizona Tucson Arizona USA; ^15^ Arizona Center for the Biology of Complex Diseases The University of Arizona Tucson Arizona USA; ^16^ Wellcome Centre of Integrative Parasitology, School of Infection and Immunity University of Glasgow Glasgow UK

**Keywords:** asthma, children, prevention

## Abstract

This report is a summary of the presentations given at the European Respiratory Society's Research Seminar on Asthma Prevention. The seminar reviewed both epidemiological and mechanistic studies and concluded that; (i) reducing exposure of pregnant women and children to air pollution will reduce incident asthma, (ii) there are promising data that both fish oil and a component of raw cow's milk prevent asthma, and (iii) modulating trained immunity by either mimicking helminth infection or oral and sublingual bacterial products is a promising area of research.

## 1 Background

Asthma is the most prevalent chronic disease in children. Preventive approaches in children before the onset of disease (primary prevention), or after first signs of disease in children at risk (secondary prevention), would be a cost‐effective way of reducing the global burden of asthma symptoms, with benefits throughout the lifecourse. This report is a summary of the presentations given at the European Respiratory Society's (ERS) Research Seminar on Asthma Prevention held in Berlin in 2023. In this Seminar, faculty from academia and industry, basic research, and early career researchers were brought together to review whether there is already sufficient scientific evidence for implementation for interventions, and how best to move the field towards novel prevention strategies. Presentations considered insights provided by epidemiological studies, potential mechanisms to be targeted, and reviewed the most promising interventions.

### 1.1 Strengthening Causal Inference from Observational Studies

Progress in the primary prevention of childhood asthma has been slow. We rely on epidemiological studies of modifiable risk factors in early life to inform the design of randomized controlled trials (RCTs) and preventive strategies. However, if evidence from observational studies is flimsy, RCTs are likely to fail. Indeed, until recently, most prenatal and postnatal interventions had been unsuccessful [1]. The harsh reality is that many published asthma‐risk factor associations in observational studies are unlikely to be causal, having arisen through chance, bias, or confounding. Another factor muddying the epidemiological waters is that studies of asthma risk factors have used an extraordinary multitude of asthma phenotypes [2]. So how can we be more rigorous in epidemiological studies and strengthen causal inference? Recent guidance on how best to control for confounding is very welcome [3]. Family based designs can be used to confirm or rule out unmeasured confounding; for example, the use of negative parental controls is especially useful with respect to intrauterine risk factors [4, 5]. Demonstration of a priori, biologically plausible, gene‐environment interactions may shed light on mechanisms and inform personalized prevention strategies. Whilst few such interactions reported in the literature have been replicated, let alone in the same paper, there are exceptions [6]. Mendelian randomization, a form of instrumental variable analysis, whereby a genetic variant is used as an unconfounded proxy for a specific exposure of interest, has the potential to confirm or refute the causality of previous observational associations [7]. However, it requires a suitable polymorphism to be available, core assumptions to be satisfied, and a study sample of tens of thousands [5]. De novo RCTs in early life are costly and time‐consuming, and children must be followed up until 5 or 6 years of age to distinguish preschool wheeze from asthma. Whilst vitamin D supplementation in pregnancy reduced preschool wheeze [8], it did not reduce later asthma in the offspring [9, 10]. By contrast, a recent trial which found that vitamin C supplementation in pregnancy reduced the risk of childhood wheezing associated with maternal smoking should give us cause for optimism that the primary prevention of childhood asthma may be achievable, at least in certain risk groups [11]. Follow up of the offspring beyond 5 years of age in this trial is eagerly awaited. How might we obtain faster randomized evidence? One way is by using epidemiology to inform animal experiments [12]. Another is to identify previous RCTs, originally undertaken with other outcomes in mind, in which randomized modification of early life exposures of interest (e.g., nutrient intake) has already occurred; children can then be followed up to measure asthma, either by questionnaire or record linkage. Using this “short‐cut” approach, the benefit of fish oil supplementation in pregnancy on offspring asthma risk was confirmed by Olsen et al. [13]. eight years before the recent Danish de novo trial described below. In summary, by adopting methodologies described above to strengthen causal inference, we hope to go down fewer blind alleys and hasten progress towards the primary prevention of asthma. The most convincing evidence for causality will come from a “triangulation” approach, whereby data are integrated from different types of study (e.g., cohort, Mendelian randomization, and trial) and similar conclusions are reached [14, 15].

### 1.2 Trained Immunity

Trained innate immunity (TI) and tolerance are two arms of memory of innate immune cells demonstrated in monocytes/macrophages, dendritic cells (DC), NK cells, stem cells and others [16]. Innate immunity is defined as augmented response on a secondary challenge with the same or different stimuli such as bacterial or viral infection, or other environmental exposure, whereas trained tolerance shows diminished response upon the secondary challenge [16]. Importantly this response from the cells of innate immunity compartment may persist even months or years, especially when hematopoietic stem cells are trained in addition to the tissue cells. Mechanistically, trained memory can be induced by special epigenetic alterations and/or with special metabolic reprogramming [17]. For instance, acquisition of histone 3 lysine 27 acetylation (H3K27ac) marks at distal enhancers, histone 3 lysine 4 methylation (H3K4me1) and consolidation of histone 3 lysine 4 trimethylation (H3K4me3) marks at the promoters of stimulated genes [16], accumulation of fumarate [18], or α‐ketoglutarate [19], are signatures of trained memory acquired by innate immune cells.

There are several epigenetic marks and metabolic cues reported in various innate immune cells, such as innate lymphoid cells (ILCs), monocytes/macrophages and DCs in adult patients with asthma of various endotypes and phenotypes, and in animal models of asthma [20, 21, 22, 23, 24]. Their responses and repertoire differ in numbers, phenotype and function from cells of healthy individuals, which might suggest innate memory, but the detailed mechanisms have not been demonstrated [25, 26]. Some treatment approaches in allergy and asthma may modify these phenomena, although mechanisms are also not well defined. Monocytes, ILCs and DCs in allergic patients acquire less pro‐inflammatory and more suppressive repertoire and phenotype upon treatment with allergen immunotherapy (AIT), as a sign of trained tolerance up to 3 years after initiation of treatment [27, 28, 29]. Similarly, gene expression, epigenetic marks, metabolic reprogramming and cellular composition of some structural cells, such as bronchial epithelial cells of patients with asthma differ significantly from healthy epithelium at the steady state, upon type 2 and non‐type 2 inflammation and after viral infections [30, 31, 32, 33, 34, 35]. Interestingly, AIT increases expression of type I/III interferons (IFN) in bronchial epithelial cells of patients with asthma upon stimulation with poly (I:C) and decreases expression of interleukin (IL)‐33, a potent epithelial alarmin [36]. This pattern is compatible with induction of trained memory in airway epithelium, and correlates with the decreased frequency of viral‐induced exacerbations in patients with asthma on AIT treatment [37, 38].

It is not well understood when these described above alterations happen, what are the initiating factors, how long they persist, to which extent they are involved in the clinical outcomes of asthma, and if they can be reversed. Several preclinical studies [39, 40, 41], birth cohorts [42, 43] and mechanistic environmental studies [44, 45, 46] suggest initiation of correct trained innate memory already in utero and/or in early life by acquiring of healthy gut, lung and skin microbiome during the so called “window of opportunity” [46]. Any disturbance of this process, exposures to deleterious viruses, allergens, bacteria, antibiotics, cigarette smoke and other environmental cues might alter the training of innate immunity and lead to development of asthma. However, mechanistic, prospective studies in humans are needed to study these phenomena in more detail. Importantly, data available so far suggest that innate memory might be modulated either in early childhood as an asthma prevention strategy [47, 48] or later in life as a treatment approach [47, 48] but more studies are needed to explore this opportunity.

### 1.3 Mechanistic Insights from a Birth Cohort

The trajectory to childhood asthma is thought to begin in utero and then progress under the influence of post‐natal exposures. The role of the maternal prenatal immune status in shaping this trajectory was recently highlighted by work in the Infant Immune Study (IIS), a birth cohort of mother‐child dyads sampled pre‐, peri‐, and post‐natally over a decade [49]. A decreased ratio of IFN‐γ to IL−13 (IFN‐γ:IL‐13) secretion by mitogen‐stimulated maternal peripheral blood mononuclear cells isolated during the third trimester of pregnancy was associated with increased prevalence of childhood asthma [50]. This relation was limited to pregnancy and was specific to non‐asthmatic mothers.

A subsequent epigenome‐wide study in a subset of IIS neonates revealed distinct DNA methylation profiles in cord blood mononuclear cells of children who did or did not become asthmatic by age 9 years [51], suggesting that perinatal epigenetic regulation of immunity acts as a gatekeeper for the asthma trajectory from birth to childhood. Since perturbations of early life gut microbiota development are linked to increased risk of asthma in childhood [52], it has been hypothesized that prenatal immune dysfunction associated with increased childhood asthma risk alters neonatal immune training and promotes early‐life airway colonization by “asthmagenic” microbiota [53]. To test this, the IIS birth cohort was analyzed for epigenetic, immunologic, and microbial features in neonates born to non‐asthmatic mothers. Results show that a module of differentially methylated CpG sites was associated with childhood asthma (but not atopy) and was enriched for microbe‐responsive elements. In vitro cytokine responsiveness to microbial products was impaired in cells from neonates born to mothers with the lowest IFN‐γ:IL‐13 ratio, suggesting that neonatal innate immunity was defective in infants who developed asthma during childhood. These infants also exhibited a distinct upper airway microbiota development characterized by early life colonization by *Haemophilus* that transitioned to a *Moraxella*‐dominated microbiota by age 36 months [54]. Overall, these findings are compatible with the concept that maternal prenatal immune status shapes the child's trajectory to asthma by altering the epigenome and trained immunity in the neonate, and then promoting pathologic upper airway microbial colonization in early life. The selective association between neonatal methylation and childhood asthma but not atopy points to a distinct epigenetic path to asthma that is independent of allergy—a notion supported by previous epidemiological work [50]. As importantly, the epigenetic network associated with asthma development in at‐risk neonates born to mothers with low IFN‐γ:IL‐13 was enriched in differentially methylated CpG sites that mapped to microbe‐responsive elements. Consistent with a role of these epigenetic modifications in innate immune function, monocyte‐derived IL‐6 and tumor necrosis factor (TNF) production in those neonates was reduced upon stimulation with bacterial LPS. Decreased innate cytokine responses to microbial pro‐inflammatory triggers point to an impairment of TI (see above). Overall, these data provide the first population‐based link between TI at birth and asthma during childhood and identify a network of epigenetically modified genes associated with decreased innate responsiveness to microbes, asthmagenic upper airway bacterial colonization in early life, and increased risk of asthma during childhood.

### 1.4 Bacterial Lysate

OM‐85 (Broncho‐Vaxom) is a lysate of 21 strains originating from pathogenic respiratory bacteria currently used as oral prophylaxis of recurrent respiratory tract infections (RTIs) in adults and children. It is a standardized complex mix of microbial content obtained by chemical lysis and comprising pathogen‐associated molecular patterns (PAMPS). Data from non‐disease animal and cell models suggest that OM‐85, via effects on mucosal cells, induces a broad non‐specific cellular and polyclonal anti‐microbial immune response with a “pre‐alert” state primarily involving the priming of DCs [55, 56, 57]. In a disease context, OM‐85 has demonstrated a capacity to attenuate infection in influenza, rhinovirus, severe acute respiratory syndrome coronavirus 2 (SARS‐CoV‐2) and respiratory syncytial virus (RSV) models [55, 56, 58, 59, 60]. A recent study has found that oral OM‐85 in a pregnant mouse model reduces the risk for fetal loss/growth restriction via, in part, enhancing myelopoiesis and programming DC function and survival, with promotion the enrichment of genes from the type I IFN pathway suggesting induction of TI [60, 61, 62]. In a study in children, Sly et al. [63] found protection against severe lower RTIs by OM‐85. After 12 months a mechanistic study using peripheral blood mononuclear cells [64], demonstrated gene network changes, in particular upregulation of IFN signaling, accompanied by network rewiring resulting in increased coordination of TLR4 expression with IFN pathway‐associated genes, again pointing towards controlling of inflammatory response intensity and duration. Finally, in a study using primary human airway epithelial cells, multiple cellular readouts found a direct effect on OM‐85 [65], and has led the development of an intranasal route preparation. Of relevance to allergic airway inflammation, repeated intranasal administrations of OM‐85 prior sensitization prevents OVA‐ and Alternaria‐induced experimental allergic via actions on DCs, Th2 responses, and the ILC type‐2/IL‐33 axis [66]. Ongoing (unpublished) studies of OM‐85 in a papain murine model of asthma lacking sensitization, found that OM‐85 administration before papain blunts allergic response caused by subsequent papain challenge 40 days later indicative of imprinting. In summary, these recent data provide mechanistic support for ongoing studies of OM‐85 as a way of preventing asthma.

### 1.5 Polybacterial Vaccine

As discussed in the section above, induction of TI is a potential therapy for asthma treatment and prevention. TI‐based vaccines (TIbV; vaccine formulations promoting TI) contain two main constituents: (i) TI inducers (frequently PAMP targeting PRR); (ii) the specific components (the antigens associated with the pathogens acting as TI‐inducers) to which adaptive immunity is aimed. Among TIbVs, MV130 is a whole heat‐inactivated polyvalent bacterial formulation consisting of 90% of Gram‐positive bacteria [67]. All these components are entire heat‐inactivated bacteria, which likely confers its capacity to induce TI. Initial studies demonstrated that long‐term sublingual administration of MV130 significantly reduced the rate of infections in recurrent RTIs patients [68]. MV130 acts on DCs enhancing their capacity to generate potent Th1, Th17 and IL‐10‐producing T cells thought Toll‐like receptor (TLR)‐ and nucleotide‐binding oligomerization domain‐containing protein (NOD)‐like‐mediated signaling pathways [67]. Studies have also demonstrated in vivo that MV130 sublingual immunization of mice also confers potent Th1, IL‐17, and IL‐10 responses against the unrelated antigen OVA, suggesting that MV130 might well induce TI [67]. And a randomized, double‐blind, placebo‐controlled phase III clinical trial has found that sublingual administration of MV130 is a safety treatment to prevent recurrent wheezing in children [69]. Interestingly, MV130 confers protection at least 6 months after treatment discontinuation, thus pointing out to long‐lasting TI mechanisms [69]. Supporting these data, mice models have found that MV130 protects against viral infections by mechanisms depending on TI via specific metabolic and epigenetic reprogramming of innate immune cells and hematopoietic precursors [70]. These in vitro and in vivo models and clinical trials show that sublingual vaccination with the whole‐heat‐inactivated vaccine MV130 induces TI and protects against recurrent infections triggered by viruses. Although, the potential capacity of MV130 to prevent or treat airway eosinophilic allergic inflammation remains, to date, unclear, unpublished data presented in the ERS research seminar suggests that MV130 reduces airway inflammation in an HDM‐induced eosinophilic asthma model. Future studies are therefore warranted aimed to further unravel the underlying molecular mechanisms involved in such anti‐allergic and anti‐eosinophilic properties. Similarly, future clinical trials investigating the potential capacity of early interventions with sublingual MV130 in children, or even at the prenatal period by treating during pregnancy, to prevent recurrent infections and allergic sensitization might be of great interest to shed light into the potential capacity of this whole heat‐inactivated mucosal formulation to prevent later asthma development in children.

### 1.6 Mimicking Parasitic Helminths

The impact of infections on inflammatory disorders has been intensively examined, some pathogens (particularly viruses) being implicated in initiating exacerbations, while others may be protective. In the latter category, parasitic helminths (worms) have proven particularly interesting [71]. At the epidemiological level, a negative correlation is observed between helminth infection and development of allergic reactivity, as found for example in schoolchildren in West Africa, in whom anthelmintic clearance of parasites increased incidence of allergic reactivity [72]. The inverse relationship between infection and allergy has been reproduced in animal models, further demonstrating causality: mice infected with common helminth parasites such as the intestinal worm *Heligmosomoides polygyrus* show suppression of airway allergic inflammation, and are protected from other pathologies such as murine inflammatory bowel disease [71]. Currently studies are seeking to identify defined products which can be transformed into new pharmacological agents via an analysis of the mechanisms whereby these agents dampen inflammation. To date, specific protein products from helminths which act to forestall allergic reactivity have been identified. Earlier work discovered a molecule that targets the alarmin interleukin IL‐33, the spark which initiates the Th2 allergic response in the tissues [73]. A more extensive set of parasite products have also been found which act to mimic the mammalian immune‐suppressive cytokine transforming growth factor (TGF) β. This cytokine is a key mediator in switching T lymphocytes from the effector mode to a regulatory (suppressive) mode; activation of the TGF‐β pathway by the parasite is beneficial by dampening host immunity, but also has bystander effects against third party antigens such as allergens, thus offering a mechanistic pathway for how parasites may protect against allergies. The family of TGFβ mimics (TGMs) represent a particularly striking example of convergent evolution, as they ligate host TGF‐β receptors despite bearing no sequence similarity to the mammalian cytokine [74]. Moreover, they also bind co‐receptors such as CD44, allowing the parasite protein to preferentially target immune cells for down‐modulation. These proteins act in animal models to prevent airway allergy, whether given at the sensitization phase or administered during airway challenge with allergens such as Alternaria, house dust mite (HDM), or ovalbumin [75], and can also dampen inflammation in the intestinal tract during models of colitis. Thus, parasite products can offer new possibilities for anti‐inflammatory therapies that are targeted at key players in the immune response.

### 1.7 Allergens

Exposure to inhalant allergens (e.g., HDM, cat, and dog allergens) is associated with the development of allergen‐specific sensitization and asthma, but their relationship is influenced by other environmental exposures (such as microbial exposure) and the genetic predisposition of the individual [76]. Understanding these inter‐relationships is an essential step for disease prevention. Interactions between environmental exposures, in addition to route and timing of exposure, together with the genetic predisposition of the host, all contribute to the complexity. It is perhaps not surprising that some studies reported that early‐life dust mite allergen exposure increases the risk of mite sensitization and asthma, while others have not confirmed these association [76]. The impact of exposure to cat and dog allergens has been extensively investigated, but also with inconsistent results. For example, several birth cohorts observed a linear dose–response relationship between cat allergen levels measured in homes in early life and increased risk of sensitization to cat in pre‐school/early school age. In contrast, cross‐sectional studies in older children and young adults reported that very high Fel d 1 levels may protect against cat sensitization [77]. Data on the effect of dog ownership is more consistent, with most (although not all) studies suggesting that having a dog in early life is protective against sensitization to dog, but also sensitization to other allergens and asthma. This suggests that protective effect of dog ownership is likely due to an environmental exposure for which dog ownership is a proxy (such as higher microbial exposure and/or diverse external microbiome). A common notion is that most exposure to inhalant allergens occurs via inhalation (and to food allergens via ingestion), sensitization may also develop because of allergen presentation through an impaired skin. Relevant to this are findings that exposure to mite allergen in infancy is associated with increased risk of mite sensitization in children with filaggrin (FLG) mutations and not in those without [78]. However, it is also of note that the modifying effect of FLG mutations is higher in early childhood, and gradually reduces over time. A totality of epidemiological evidence suggests that the effect of interventions to alter allergen exposure to impact the development of sensitization and asthma is likely to differ between children with different genetic predisposition and will be influenced by other concomitant environmental exposures (primarily bacterial), indicating that only individuals with a specific genetic susceptibility and exposome may benefit from any particular interventions (either avoidance or high‐level exposure). Clinical outcomes of the primary prevention studies which tested the effectiveness of allergen avoidance in pregnancy and early life on subsequent development of sensitization and asthma published to date are inconsistent [79, 80, 81]. Therefore, there is no evidence‐base for the use of allergen avoidance for the primary prevention of these conditions, and more nuanced analyses are required before we can draw definitive conclusions and give any meaningful advice.

### 1.8 Fish Oil

Dietary changes in westernized countries have resulted in an increase in the intake of *n*−6 polyunsaturated fatty acids and a decrease in the intake of *n*−3 polyunsaturated fatty acids, especially the long‐chain polyunsaturated fatty acids (*n*−3 LCPUFA)—eicosapentaenoic acid (EPA) and docosahexaenoic acid (DHA)—found in oils from cold‐water fish. There is considerable evidence from observational human studies and animal studies that low levels of *n*−3 LCPUFA during pregnancy might increase the risk of atopic disorders in the offspring, including asthma, allergy and atopic dermatitis, but the evidence from randomized clinical trials (RCTs) of fish oil supplementation has been inconsistent [82]. An RCT of fish oil supplementation during pregnancy in 736 Danish women found that supplementation in the last trimester of pregnancy reduced the risk of asthma in the offspring by 30% by age 5 years [83]. This effect was most pronounced in mothers with low EPA and DHA blood levels before the intervention, and in mothers who carried fatty acid desaturases (FADS) gene risk variants associated with lower levels of EPA and DHA. Evidence of a protective effect was also found for RTIs and gastroenteritis, while there was no effect on allergy or atopic dermatitis. Surprisingly, supplementation with fish oil was associated with an increased body mass index (BMI) during childhood [84]. These results need to be replicated in future large‐scale trials to provide sufficient evidence base for recommendations on fish oil supplementation during pregnancy. Future studies should address the unexpected protective effects against infections, and potential modifying factors, such as maternal EPA and DHA blood levels, genotype, lifestyle, dietary factors, and ethnicity. Also, potential adverse effects should be investigated including metabolic consequences of a potential increase in childhood BMI. If the beneficial effects on early asthma and infections are confirmed, fish oil supplementation during pregnancy could be one of the first preventive measures for these common childhood disorders, with large health benefits in offspring of high‐risk mothers. From a research perspective, this could also provide insight into the still poorly understood mechanisms of infection susceptibility in childhood.

### 1.9 Air Pollution

It is now well established that childhood exposure to air pollution has adverse effects on respiratory health including the development of asthma [85]. To date, much of the focus of research, especially in the global north, has been on outdoor air pollution and associations between exposure at the home address and respiratory health. Such evidence is used to support action taken by local and regional authorities to improve ambient air quality. Although such improvements will have benefit for all, they are based on ecological models, without consideration of individual vulnerabilities and little or no agency given to those who are most affected. Furthermore, changes in ambient air quality may take decades to have significant impact. Recent advances in wearable sensing technology, has led healthcare professionals and researchers to seek robust, personalized and actionable recommendations to help protect the mother and fetus and the child from inhaled toxins. However, this personalized approach to exposure reduction in clinical settings presents significant challenges. For example, personal exposure monitoring studies using relatively expensive devices have demonstrated that heterogeneity in exposure to toxic air is driven not only by residential address but by proximity to diverse sources indoors and outdoors, while at school, home, and other locations, and variables such as mobility, ventilation, cooking, and second‐hand smoke exposure [86]. Thus, a personalized approach to exposure provides increased opportunities for mitigation not possible with an ecological approach [87]. But formulating tailored advice remains difficult without more detailed information than is currently routinely available. The use of affordable wearable technologies that can be integrated into everyday life, such as a school bag that also monitors air pollution [88], is one solution, but management and interpretation of data remains problematic without automated algorithms that can transform time series data streams into easily interpretable and actionable advice. A further challenge is the acceptability, transferability, and appropriateness of such advice. In common with many other public health challenges, changes in behavior require the target to feel that they have the capability, opportunity, and motivation to change [89]. These characteristics will be vastly different across demographics and geographies and need to be co‐developed with the target community. Thus, there remains an urgent need for evidence of the efficacy of this approach as a public health improvement strategy, with control of asthma being a high priority target population.

### 1.10 Milk

It has been repeatedly shown that children living on farm are at lower risk of asthma and allergies. This “farm effect” has partially been explained by consumption of raw cow's milk. A meta‐analysis on 8 pertinent studies confirmed the protective effect of raw milk consumption early in life on asthma (odds ratio [OR], 0.58; 95% CI, 0.49 to 0.69), current wheeze (OR, 0.66; 95% CI, 0.55 to 0.78), hay fever or allergic rhinitis (OR, 0.68; 95% CI, 0.57‐0.82), and atopic sensitization (OR, 0.76; 95% CI, 0.62 to 0.95) [90]. The effect on asthma was similarly observed in farm children (OR, 0.62; 95% CI, 0.58 to 0.82) and in children living in rural areas but not on a farm (OR, 0.60; 95% CI, 0.48 to 0.74). This phenomenon suggests first that the effect of farm milk consumption is independent of other farm exposures and second that children not living on a farm may essentially profit from this effect. Nevertheless, there is a minimal residual risk of life‐threatening infections. Therefore, consumption of raw milk and products cannot be advised. Raw farm milk and industrially processed milk differ in many aspects such as removal of cellular components, manipulation of the fat fraction, and various degrees of heating. Preliminary evidence attributes the effect to heat‐labile molecules and components residing in the fat fraction [91, 92]. In particular, iron‐binding proteins such as lactoferrin may keep detrimental gut bacteria at bay as illustrated by a low asthma prevalence in children using milk as a protein source rather than meat [93]. Moreover, raw milk fosters adequate maturation of the gut microbiome, which is involved in mediating the protective effect on asthma [94]. The Milk Against RTIs and Asthma (MARTHA) trial (www.martha-studie.de) is currently testing the feasibility of an interventional study with pasteurized but otherwise unmanipulated natural full‐cream cow's milk. The MARTHA trial randomized 260 children of whom 221 completed the intervention (85%). Four children had to be excluded retrospectively for exclusion criteria and 35 families withdrew their consent during the study or were lost to follow‐up. The participants were followed for an average duration of 104 weeks, that is, 2 years. Altogether, they completed 22,988 weekly surveys recording information on milk consumption and symptoms suggestive of adverse events and infections. Two percent of surveys triggered a specific follow‐up for potential adverse events by trained study physicians, but no case of milk allergy was evidenced by a clinical work‐up including food challenge. An early interim analysis of the first participants suggested a favorable effect on the maturation of the gut microbiome. Respiratory tract infections were difficult to assess due to a reduced incidence during lockdown periods because of the SARS‐CoV‐2 pandemic, which also interfered with recruitment speed and completeness of clinical visits. Ultimately, a definitive trial for the beneficial effects of minimally treated cow's milk is urgently needed. For addressing the most relevant outcome, that is, childhood onset asthma at age 5 to 6 years, a samples size of 2700 children would be necessary. If such a RCT was positive, it would provide an effective prevention strategy for asthma and allergies, which could be easily and sustainably implemented in everyday life.

### 1.11 Limitations

A limitation of this ERS research seminar was that it did not review all potential interventions such as the ongoing RCT of omalizumab for preventing asthma in high‐risk children [95]. But some feasible interventions for primary prevention were identified. First, bacterial products are a promising since they are already available for clinical use and results of the ongoing primary and secondary prevention trials are awaited (Table 1). Second, the long‐speculated possibility that the mimicking helminth infection may provide novel therapies has potential, but a therapeutic intervention is not yet available to test. Third, the evidence base remains weak for the primary prevention of asthma by allergen avoidance. Fourth, there are promising data for fish oil which should be assessed in a definitive RCT. Fifth, there is a compelling case to reduce road traffic emissions to reduce incident asthma, albeit how individuals can mitigate their own exposure remains unclear. Finally, although there are promising data to suggest that a component of raw cow's milk prevents asthma, and more mechanistic studies are needed since what component to test in a RCT remains unclear. In conclusion, given the potential public health benefits of primary prevention of asthma, funders should consider supporting high risk/high reward clinical studies in this area, especially using therapeutic interventions whose side effects are well known.

**Table 1:** Summary of potential options for primary prevention of asthma that were discussed at the ERS research seminar.

Intervention	Support from relevant mechanistic studies	Support from epidemiological studies	Support from clinical trials	Investigational Medical Product (IMP) or Intervention currently available
Bacterial lysate OM85	Yes	No	Primary prevention trials in progress	Yes
Polyvalent bacterial vaccine MV130	Yes	No	No primary prevention trials	Yes
Helminth mimic	No	Yes	No primary prevention trials	No
Allergen exposure mitigation	No	No	Inconsistent results from primary prevention trials	Yes
Fish oil supplementation	No	Yes	Yes	Yes
Air pollution mitigation	Yes	Yes	Trials currently not feasible	Yes
Milk product intervention	Yes	Yes	No primary prevention trials	No

## Author Contributions

Conception: J.G. and E.v.M. Manuscript drafting and revising: J.G., B.B., K.B., A.C., M.E., C.P., O.P., S.S., M.S., D.V., R.M., E.v.M. All authors contributed to writing the final manuscript. All authors have read and approved the final version of this manuscript.

## Conflicts of Interest

The research seminar was supported by a grant from the European Respiratory Society. J.G. *Support*; NIHR Senior Investigator Award, *Grants*; OM Pharma, Marinomed; *Advisory board*; OM Pharma, Omron, *Honoraria*; AstraZeneca, *Equipment and materials donation*; OM Pharma, Immunotek S.L., Omron, *Payment for medical evidence*; Hogg Robertson Solicitors. K.B. *Honoraria*; Sanofi and AstraZeneca, *Meeting support*; ALK‐Abelló Nordic, *Advisory Board*; ALK‐Abelló Nordic. A.C. *Grants*: Medical Research Council, EPSRC, Wellcome Trust, *Consulting fees*; Sanofi, Reacta Biotech, *Honoraria*; GSK AstraZeneca, Sanofi, Stallergens‐Greer, La Roche Possay, *Board Member;* World Allergy Organization. M.E. *Grants;* German Federal Ministry of Education and Research, Dutch Longfonds, Bavarian State Ministry of Health and Care, *Patents;* EP000002361632B1, EP1964570B1, *Equipment donation;* Royal Friesland Campina. M.S. *Grants*; Swiss National Science Foundation (SNSF) nr 189334/1 and 224154, GSK, Novartis, Stiftung vorm. Bündner Heilstätte Arosa and OM Pharma; *Speaker's fee*; AstraZeneca. *Board Member*; EAACI. C.P. *Support*; Employee of OM Pharma, *Patents*; US20220143111A1, WO2020182970A, *Shares*; OM Pharma. P.O. *Grants*; UCM‐Inmunotek S.L, Comunidad Autónoma de Madrid (CAM), MICINNIN, *Consulting fees*; AstraZeneca, Pfizer, GlaxoSmithKline, Inmunotek S.L, Novartis, Sanofi‐Genzyme, *Honoraria*; AstraZeneca, Pfizer, GlaxoSmithKline, Inmunotek S.L, Novartis, Sanofi‐Genzyme, *Advisory Board*; AstraZeneca, Pfizer, GlaxoSmithKline, Inmunotek S.L, Novartis, Sanofi‐Genzyme, *Board Member*; EAACI. D.V *Grants*; National Institute of Health. E.v.M. *Grants*; German Research Foundation (DFG), German Federal Ministry of Education and Research (BMBF), German Center for Lung Research, Bavarian State Ministry of Health and Care, OM Pharma S.A, BMBF (Federal Ministry of Education and Research), European Research Council, *Royalties/licences*; Elsevier GmbH, Georg Thieme Verlag, Springer‐Verlag GmbH, Elsevier Ltd, Springer Nature Group, Deutscher Apotheker Verlag, *Honoraria*; ALK‐Abello Arzneimittel GmbH, Japanese Society of Pediatric Allergy and Clinical Immunology (JSPACI), Klinikum Rechts der Isar, University of Colorado, Paul‐Martini‐Stiftung, Astra Zeneca BioPharmaceuticals Medical, Imperial College London, Children's Hospital Research Institute of Manitoba Kompetenzzentrum für Ernährung (Kern), OM Pharma S.A. Swedish Pediatric Society for Allergy and Lung Medicine, Chinese College of Allergy and Asthma (CCAA), Abbott Laboratories, Deutscher Apotheker Verlag GmbH & Co. KG, Socieded Chilena de Enfermedades Respiratorias, Japanese Society of Allergology, British Society for Asthma and Clinical Immunology, American Academy of Allergy, Asthma & Immunology, European Respiratory Society. The remaining authors declare no conflicts of interest.

## Data Availability

Data sharing not applicable to this article as no datasets were generated or analyzed for this review.
